# Trifarotene for Facial and Truncal Acne Vulgaris: A Narrative Review of Clinical Trial Evidence

**DOI:** 10.7759/cureus.114659

**Published:** 2026-08-17

**Authors:** Jordan P Krawchuk, Kevin Tamucci

**Affiliations:** 1 Medicine, Saba University School of Medicine, The Bottom, BES; 2 Anesthesia, Saba University School of Medicine, The Bottom, NLD

**Keywords:** acne-induced hyperpigmentation, acne scars, acne vulgaris, clinical trials, efficacy, safety, tolerability, topical retinoids, trifarotene, truncal acne

## Abstract

Acne vulgaris is a common inflammatory skin disease with substantial physical and psychosocial burden. Trifarotene, a fourth-generation topical retinoid with selective retinoic acid receptor-gamma (RARγ) activity, is notable for having published clinical trial data in facial, truncal, and hyperpigmentation-associated acne. This narrative review summarizes available clinical trial evidence regarding its safety, tolerability, efficacy, and patient-centered outcomes. A focused literature review was conducted in August 2025 using PubMed to identify English-language clinical studies evaluating topical trifarotene for acne vulgaris, with a supplementary update search conducted in July 2026 to capture additional eligible literature. Eligible studies included interventional and prospective clinical studies reporting efficacy, safety, tolerability, or patient-reported outcomes in facial, truncal, or hyperpigmentation-associated acne, and findings were summarized narratively given differences in study design, treatment regimens, comparators, and reported outcomes. Five studies met the inclusion criteria, including four randomized controlled trials and one long-term prospective study. Across studies, trifarotene was generally well tolerated, with adverse events being predominantly mild. Any localized retinoid-associated skin reactions peaked early and improved over time. Trifarotene was also associated with improvement in lesion counts, investigator success rates, and selected patient-reported outcomes, including measurable benefit for acne-induced hyperpigmentation, although one trial did not meet its prespecified primary endpoint at Week 24. Short-term randomized trials demonstrated benefit over vehicle, while long-term data suggested sustained efficacy and acceptable tolerability over 52 weeks. Current clinical trial evidence suggests trifarotene may be a useful topical treatment option for moderate facial and truncal acne vulgaris, with a broader evidence base than most topical retinoids in these areas. The evidence base can be strengthened by additional studies, broader population diversity, and direct head-to-head comparisons with other topical retinoids.

## Introduction and background

Acne vulgaris is among the most prevalent dermatological conditions globally, affecting approximately 9.4% of the population and ranking as the eighth most common disease [1]. Beyond its prevalence, acne imposes substantial public health burdens, including significant treatment costs and notable psychosocial consequences such as depression, anxiety, and social stigma [2,3]. Although acne primarily affects adolescents, up to 50% of adults continue to experience symptoms, which may result in greater psychological impact due to the misconception of acne as a condition limited to teenagers [2]. These considerations point to the need for effective and durable treatment strategies that target both active acne and residual scarring.

Pathophysiology and treatments 

Comedones in acne result from follicular hyperkeratinization, excess sebum production, proliferation of Cutibacterium acnes, and inflammation of the pilosebaceous unit [2]. Acne vulgaris includes a range of lesions, from minor comedones to inflammatory nodules and cysts, varying in size and severity. Lesions are most commonly found on the face but can also occur on the chest, shoulders, and back. Truncal acne remains understudied in clinical trials despite its clinical and psychosocial burden. Current guidelines recommend a stepwise approach, starting with topical therapies for mild to moderate acne and progressing to systemic agents (isotretinoin, spironolactone, and doxycycline) for severe or refractory disease [4].

Topical retinoids are a cornerstone of acne therapy, functioning as vitamin A derivatives [5]. Retinoids normalize follicular epithelial desquamation, reduce inflammation, and prevent comedone formation [6]. Their mechanism is mediated through nuclear retinoic acid receptors (RARs), which regulate keratinocyte proliferation and differentiation [6]. Retinoids are often employed as monotherapy or in combination with antimicrobials or systemic agents. Treatment efficacy in trials is often measured using standardized scales such as the Investigator’s Global Assessment (IGA) for facial lesions and the Physician’s Global Assessment (PGA) for truncal lesions, as used in these trials.

Retinoid history 

Four generations of retinoids have been developed for topical use, and each version has variable receptor affinity. Trifarotene is the newest topical retinoid, approved by the U.S. Food and Drug Administration (FDA) in 2019 [7]. This was the first approval of a new retinoid by the FDA in 20 years [8]. Its brand name is Aklief, and its research and development code name was CD5789 [6]. It is unique in its selective agonist activity at the RAR-gamma (RARγ) receptor [7]. This RAR isoform is most abundant in keratinocytes [7]. This receptor specificity for keratinocytes is proposed to contribute to targeted local activity with limited systemic absorption [7], although this has not been directly established through comparative clinical trials against other topical retinoids.

Although trifarotene demonstrates therapeutic potential [9], its clinical evaluation remains limited to a small number of trials, primarily two identical Phase III randomized controlled studies that were explored in this review [5]. Research on trifarotene’s efficacy and safety began in 2012. These studies offer foundational evidence regarding its role in acne management.

Rationale and objective 

Trifarotene is a mechanistically distinct topical retinoid and the first retinoid with substantial clinical trial data specifically addressing both facial and truncal acne. Because truncal acne remains relatively underrepresented in the literature despite its clinical relevance, a focused narrative review of the available trial evidence is warranted. More recent trial evidence has also begun to address acne-induced hyperpigmentation. This is a further long-term sequela of acne vulgaris with particular relevance for patients with darker skin types, which this review incorporates alongside the facial and truncal evidence base. The aim of this review is to summarize the safety, tolerability, efficacy, and patient-reported outcomes associated with topical trifarotene in moderate to severe acne vulgaris, while also identifying important limitations in the current evidence base and priorities for future study.

## Review

Methods 

A focused narrative literature review was conducted using PubMed (Medical Literature Analysis and Retrieval System Online (MEDLINE)) to identify clinical studies evaluating trifarotene for acne vulgaris. The search was designed to capture human studies involving facial and/or truncal acne and was limited to English-language publications from January 2015 through July 2026, with the final search conducted on July 26, 2026, prior to submission. Search terms included “trifarotene” and “acne vulgaris,” with article-type filters used to identify clinical and prospective studies. A single database and a single reviewer were used by design, reflecting the scope and intent of a narrative review rather than a systematic review. This approach was considered appropriate given the small and well-defined body of trifarotene-specific clinical trial literature currently indexed. The original PubMed search string is reproduced below.

(“trifarotene”[Supplementary Concept] OR “trifarotene”[All Fields]) AND (“acne vulgaris”[MeSH Terms] OR “acne vulgaris”[All Fields] OR (“acne”[All Fields] AND “vulgaris”[All Fields]) OR “acne”[All Fields])

Filters limited article types to clinical trial, comparative study, controlled clinical trial, observational study, pragmatic clinical trial, evaluation study, and randomized controlled trial. Combined with the supplementary update search, this strategy yielded 53 records in total. After removal of four duplicates, 49 unique articles underwent title and abstract screening. 

To confirm that article-type filtering did not exclude eligible primary studies, the same search was subsequently rerun without article-type restrictions, yielding 70 records. Manual screening of the 17 additional records identified no further studies meeting inclusion criteria; excluded records consisted of pharmacovigilance database analyses, case reports, pharmacokinetic/safety-only studies without efficacy outcomes, studies using trifarotene as an active comparator rather than the intervention of interest, and mechanistic/transcriptomic studies without clinical efficacy endpoints.

*Inclusion and Exclusion Criteria* 

The inclusion criteria were developed using the Population, Intervention, Control, and Outcomes (PICO) framework to define the scope of the review and maintain consistency during study selection. P: Individuals diagnosed with acne vulgaris (facial and/or truncal); I: topical trifarotene in any formulation, with or without other interventions; C: placebo, vehicle, or no comparator, with noncomparative studies eligible if they provided long-term safety or efficacy data not otherwise available from comparator-controlled trials; and O: clinical effectiveness, including reduction in lesion counts and IGA or PGA success rates.

The inclusion of noncomparative studies under this criterion reflects the value of long-term, real-world tolerability data, which the comparator-controlled trials in this review did not capture given their shorter durations (12-24 weeks).

Exclusion criteria included reviews, meta-analyses, case reports, editorials, commentaries, and non-human trials. Studies without primary data, not using trifarotene, or not reporting relevant clinical outcomes were excluded. Studies outside the specified date range were also excluded.

Study Design

Primary interventional or observational studies in humans published in English.

Study Selection Process

Titles and abstracts were screened by a single reviewer, followed by full-text assessment of potentially eligible studies. At the screening stage, studies were excluded if they did not evaluate trifarotene, did not focus on acne vulgaris, were not original clinical investigations, or did not meet the predefined eligibility criteria.

**Figure 1:**
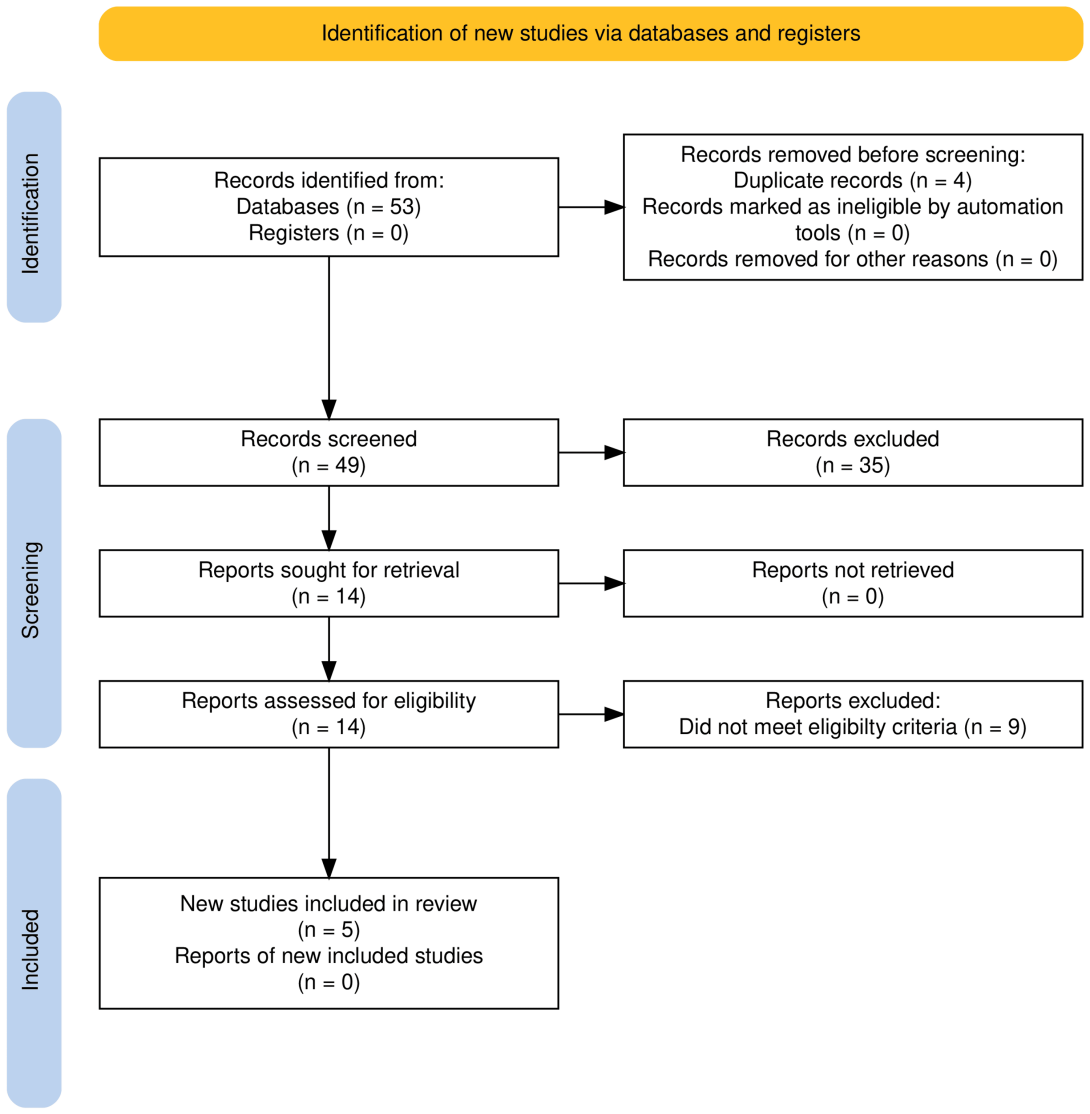
Study Selection Process Study selection flow diagram summarizing the literature search conducted on July 26, 2026 [13].

Figure 1 presents the study selection process. A total of 53 records were found. After duplicate removal, 49 unique articles remained for screening. Of these, 35 were excluded after title and abstract review, and 14 articles underwent full-text assessment. Nine were excluded at full-text review because they did not meet eligibility criteria or did not report relevant outcomes, leaving five studies for inclusion, among them Alexis et al. [10]. The diagram was generated using the PRISMA 2020 flow diagram tool [11]. The five included studies were Tan et al. [5], Alexis et al. [10], Blume-Peytavi et al. [12], Del Rosso et al. [13], and Schleicher et al. [14]. To provide a structured appraisal of internal validity, the Cochrane Risk of Bias 2 (RoB 2) tool [15] was applied to the randomized trials, and the Risk Of Bias In Non-randomized Studies of Interventions (ROBINS-I) tool [16] was applied to the noncomparative long-term study (Tables 1, 2).

**Table 1:** Cochrane Risk of Bias 2 (RoB 2) Randomized Controlled Trials Table Low: low risk; SC: some concerns; High: high risk; LOCF: last-observation-carried-forward; IGA:  Investigator’s Global Assessment; PGA: Physician’s Global Assessment; MMRM: mixed-effects models for repeated measures; ODS: Overall Disease Severity; PAHPI: Post-acne Hyperpigmentation Index Brief justifications are included in each cell. Risk of bias assessed using the Cochrane RoB 2 tool [15].

Study	Randomization Process	Deviations from Intended Interventions	Missing Outcome Data	Measurement of the Outcome	Selection of the Reported Result	Overall Bias
Tan et al., 2019 [5] (PERFECT 1 & 2)	Low: Computer-generated allocation, baseline balance, allocation concealed via identical vehicle.	Low: Double-blind, matched vehicle, no evidence of protocol deviations affecting outcomes.	Low: Low attrition and LOCF used; reasons for dropout balanced and unlikely to bias effect.	Low: Blinded investigators verified IGA/PGA and lesion counts.	Low: Prespecified endpoints and analyses aligned with protocol and publication.	Low
Del Rosso et al., 2022 [13]	Low: Central randomization with balanced groups; double dummy for blinding of systemic and topical agents.	Low: Double-blind, adherence monitored, effect-of-assignment framework maintained.	Low: Modest attrition; MMRM accommodates missingness, sensitivity consistent.	Low: Blinded assessment of IGA and lesion counts with standard procedures.	Low: Prespecified primary and secondary endpoints; statistical plan followed.	Low
Schleicher et al., 2023 [14]	Low: Within-patient side randomization and allocation of sides across patients.	SC: Participants aware of side assignment; possible differences in skin care or crossover between sides.	Low: Low dropout rate; analyses mainly on completers with sensitivity checks.	SC: Investigator outcomes blinded; patient-reported scar scales unblinded, potentially introducing bias.	Low: Outcomes and timepoints set in advance; analysis aligned with protocol.	SC
Alexis et al., 2024 [10]	Low: Parallel-group randomization with matched vehicle to maintain blinding.	Low: Double-blind; standardized skincare/photoprotection regimen applied equally to both arms.	SC: Specific attrition rates, reasons for dropout, and handling of missing data for ODS and PAHPI were not extracted from the available report; insufficient information to rate as low risk.	Low: Blinded assessment of ODS, PAHPI, and IGA using standard procedures.	Low: Primary and secondary endpoints prespecified; both positive and null results reported transparently.	SC

**Table 2:** Risk Of Bias In Non-randomized Studies of Interventions (ROBINS-I) Non-comparative Study Table Low: low risk; Mod: moderate risk; Serious: serious risk; PROs: patient-reported outcomes Brief justification included. Risk of bias assessed using the ROBINS-I tool [16].

Study	Confounding	Selection of Participants	Classification of Interventions	Deviations from Intended Interventions	Missing Data	Measurement of Outcomes	Selection of Reported Result	Overall Bias
Blume-Peytavi et al., 2020 [12]	Serious: No comparator arm; improvements might reflect natural history or placebo effects.	Low: Broad inclusion of adolescents and adults with multi-centre recruitment.	Low: Intervention (trifarotene cream daily) clearly defined and consistently applied.	Low: Open label but consistent regimen; no evidence of deviations affecting outcomes.	Mod: Attrition over 52 weeks, but most discontinuations reported with reasons described.	Mod: Investigator-assessed outcome; open-label design increases risk of expectation bias, particularly for PROs.	Low: Outcomes prespecified and consistent with protocol and publication.	Serious: Absence of a comparator and open-label design limits causal inference despite long-term value.

*Definition of Outcome Measures* 

IGA: A 5-point numerical scale that rates acne severity on the face with scores from 0 (clear/no acne lesions) to 4 (severe amounts of acne lesions)[17]. IGA success is defined as a score of 0 or 1 with at least a 2-grade improvement from baseline [17].

PGA: similar to IGA, but applied to truncal acne (chest, back, shoulders). PGA success is defined identically to IGA [17].

Scar Global Assessment (SGA): an identical grading scale used to measure atrophic scars on the face from healed acne lesions [18]. A score of 4 is defined as deep or extensive scars. SGA success is defined identically to IGA [18].

Overall Disease Severity (ODS): a clinician-rated global severity score used specifically in Alexis et al. [10] to capture combined acne and hyperpigmentation severity in a single measure, distinct from the IGA and PGA scales used in the other included trials.

Post-acne Hyperpigmentation Index (PAHPI): a hyperpigmentation-specific outcome used in Alexis et al. [10] to quantify the extent and severity of hyperpigmented lesions independently of overall acne severity, allowing the trial to separate a treatment effect on pigmentation from a treatment effect on active acne [19].

Patient Global Assessment of Change (PGAC) and the Self-Assessment of Acne Scars (SCARS) questionnaire were instruments developed specifically within Blume-Peytavi et al. [12] and Schleicher et al. [14], respectively, and were not identified as separately validated external tools.

These measurements reported by clinicians were the primary outcomes explored in multiple included studies and provide a standardized form of comparison across the research. Standardized investigator training and calibration procedures were reported in Tan et al. [5] and Del Rosso et al. [13].

*Data Extraction and Evidence Table* 

Because the included studies varied in design, comparators, treatment approach, follow-up duration, and reported outcomes, results were synthesized narratively, and no meta-analysis was performed. Definitions of the various outcomes measured across the included studies are summarized in Table 3.

**Table 3:** Definitions of clinician-reported and patient-reported outcome measures used across the five included studies.

Outcome	Definition / Description	Type
Investigator's Global Assessment (IGA) [17]	5-point scale for facial acne severity (0 = clear, 4 = severe). Success = score 0/1 with ≥2-grade improvement from baseline.	Clinician-reported
Physician's Global Assessment (PGA) [17]	5-point scale for truncal acne severity (0 = clear, 4 = severe). Success = score 0/1 with ≥2-grade improvement from baseline.	Clinician-reported
Scar Global Assessment (SGA) [18]	5-point scale for atrophic acne scars (0 = clear, 4 = severe). Success = score 0/1 with ≥2-grade improvement.	Clinician-reported
Overall Disease Severity (ODS) [10]	Composite clinician-rated severity score combining acne and hyperpigmentation severity into a single measure; used only in Alexis et al. [10].	Clinician-reported
Post-acne Hyperpigmentation Index (PAHPI) [19]	Hyperpigmentation-specific score quantifying extent and severity of hyperpigmented lesions, independent of overall acne severity; used only in Alexis et al. [10].	Clinician-reported
Dermatology-related quality of life in adults (DLQI) or children (CDLQI) [20, 21]	10-item questionnaires assessing DLQI or CDLQI. Higher scores = worse quality of life (QoL).	Patient-reported
Acne-QoL [22]	Acne-specific questionnaire measuring psychosocial impact across domains such as self-perception, role-emotional, and social functioning.	Patient-reported
Patient Global Assessment of Change (PGAC) [12]	Single-item measure of patient's overall perception of improvement since baseline.	Patient-reported
Self-Assessment of Acne Scars (SCARS) [14]	Patient-reported questionnaire quantifying scar visibility and impact on self-image.	Patient-reported

For each included study, data were extracted into a standardized evidence table summarizing publication year, study design, population, intervention details, efficacy outcomes, adverse events, and patient-reported outcomes (Table 4).

**Table 4:** Summary of study design, population, intervention, and key efficacy and safety outcomes for the five included trials. AE: adverse event; TEAE: treatment-emergent adverse event; IGA: Investigator’s Global Assessment; PGA: Physician’s Global Assessment; SGA: Scar Global Assessment; ODS: Overall Disease Severity; PAHPI: Post-acne Hyperpigmentation Index; QoL: quality of life. All five included trials were funded by Galderma R&D, the manufacturer of trifarotene; author disclosures across these trials commonly include consulting, speaking, or investigator relationships with Galderma.

First Author	Year	Study Design	Study Population	Therapy / Exposure	Outcome / Results
Tan et al. [5]	2019	Two identical, multi-centre, randomized, double-blind, vehicle-controlled Phase III trials (PERFECT 1 & 2, 12 weeks)	>2,400 patients ≥ 9 years with moderate facial and truncal acne	Trifarotene 50 μg/g cream once daily vs vehicle	Week 12: IGA success 29.4–42.3% vs 19.5–25.7% vehicle (p<0.001). PGA success 35.7–42.6% vs 25.0–29.9% (p<0.001). Significant lesion reductions (−19 to −30 vs vehicle, p<0.001). Mild local AEs peaked Weeks 1–2; discontinuation <2%.
Del Rosso et al. [13]	2022	Multi-centre, randomized, double-blind, parallel-group study (12 weeks)	202 patients ≥ 12 years with severe facial acne (IGA 4)	Trifarotene 50 μg/g cream + doxycycline 120 mg vs vehicle + placebo	Week 12: total lesions −69.1 vs −48.1 (p<0.0001). IGA success 31.7% vs 15.8% (Week 12, p=0.0107). Patient satisfaction 86% vs 54%. TEAEs ~13%.
Schleicher et al. [14]	2023	Phase IV, multi-centre, randomized, double-blind, vehicle-controlled split-face trial (24 weeks)	121 patients, ages 17–34, moderate-severe acne with ≥10 atrophic scars per face	Trifarotene 50 μg/g cream once daily (one side) vs vehicle (contralateral side)	Week 24: mean scar reduction −5.9 vs −2.7 (p<0.0001). SGA success 31.3% vs 8.1%. IGA success 63.6% vs 31.3%. Patient-reported scar improvement 49% vs 37%.
Blume-Peytavi et al. [12]	2020	Multi-centre, open-label, long-term Phase III safety and efficacy trial (52 weeks)	453 patients aged 9–54 with moderate-severe facial and truncal acne	Trifarotene 50 μg/g cream once daily (face + trunk)	Week 12: IGA 26.6%, PGA 38.6%. Week 52: IGA 65.1%, PGA 66.9%; combined success 57.9%. QoL 'no effect' rose 22.6% to 53.8%. TEAEs 12.6%, discontinuation 2.9%.
Alexis et al. [10]	2024	Multi-centre, randomized, double-blind, vehicle-controlled Phase IV trial with standardized skincare/photoprotection (24 weeks)	123 patients ages 13–35 with acne vulgaris and risk of acne-induced hyperpigmentation; ~70% Fitzpatrick skin types IV–VI	Trifarotene 50 μg/g cream once daily + skincare regimen vs vehicle + skincare regimen	Week 12: ODS −1.6 vs −1.1 (p=0.03). Week 24 (primary endpoint): ODS −2.1 vs −2.1 (p=0.8879, not met). PAHPI −18.9% vs −11.3% (Week 24, significant). High patient satisfaction/adherence. Favourable safety profile across skin types.

Durations of treatment, regimen details, and patient follow-up (both primary and secondary endpoints), along with any adverse event profiles, were also recorded.

The evidence table (Table 4) was created to display key characteristics of the five included studies. Age ranges were taken directly from each report. When percentage improvement was not explicitly presented, it was calculated from baseline and follow-up counts using the following equation: Percent reduction = (baseline count − follow-up count) / baseline count.

Percentages calculated by the review authors using this formula are distinguished in the text from percentages directly reported by the original study.

Numeric data were uniformly included in the table. Tan et al. [5] reported Week 12 IGA and PGA success on face and trunk, along with percentage reductions in lesion counts (Table 4).

Blume-Peytavi et al. [12] provided the success rates for IGA and PGA at weeks 12 and 52, which were used to calculate overall improvement. Blume-Peytavi et al. [12] also had patient-reported outcomes, including Investigator Global Assessment of Change (IGAC) and Patient Global Assessment of Change (PGAC) [12], as well as Quality of Life (QoL) using the Dermatology Life Quality Index (DLQI) [20] for adults and Children’s DLQI (CDLQI) for patients aged <16 years [21].

Del Rosso et al. [13] reported absolute and percentage changes in lesion counts, and mean percent improvements were included, along with IGA change data. Patient-reported outcomes included the Acne-Specific Quality of Life (Acne-QoL) [22] questionnaire, patient satisfaction, and drug acceptability. Because this trial evaluated trifarotene in combination with doxycycline against vehicle plus placebo rather than trifarotene alone, its results should be interpreted as evidence for the combination regimen. The independent contribution of trifarotene cannot be isolated from this design as mentioned in the Discussion section.

Schleicher et al. [14] reported the mean reduction in scar count and success rates, including IGA and PGA, with patient-reported outcomes captured via the SCARS Questionnaire [14].

Alexis et al. [10] reported ODS scores and a PAHPI at Weeks 12 and 24, alongside acne lesion counts and IGA, in a population enrolled specifically for risk of acne-induced hyperpigmentation. Patient satisfaction and adherence with an accompanying skincare regimen were also reported. Full results appear in Table 4, with the risk-of-bias appraisal in Table 1.

Most patient-reported findings were summarized qualitatively because the outcomes and reporting methods were not uniform across studies. These findings were still considered important because they help place lesion reduction in a broader clinical context.

Results

Classification of the Study Design 

All the selected primary studies were randomized controlled trials, except for Blume-Peytavi et al. [12], which was an open-label, noncomparative study. Throughout all studies, participants ranged in age from nine to 54 years, and all evaluated the effects of topical trifarotene 50 μg/g cream applied once daily for the treatment of moderate to severe acne vulgaris. Tan et al. [5] conducted a multi-centre, randomized, double-blind, vehicle-controlled Phase III trial (comprising two independent studies). Del Rosso et al. [13] conducted a randomized, double-blind, parallel-group trial assessing combination therapy (trifarotene + doxycycline). Schleicher et al. [14] conducted a multi-centre, randomized, double-blind, vehicle-controlled split-face trial. The 52-week Blume-Peytavi et al. [12] study was an open-label, noncomparative, multi-centre Phase III safety and efficacy study. Alexis et al. [10] conducted a multi-centre, randomized, double-blind, vehicle-controlled Phase IV trial over 24 weeks, evaluating trifarotene alongside a standardized skincare and photoprotection regimen in patients with acne vulgaris and risk of acne-induced hyperpigmentation; a full population breakdown, including Fitzpatrick skin type distribution, appears in Table 4.

Randomization and Blinding Processes

Randomization in Tan et al. [5] and Del Rosso et al. [13] was computer-generated and stratified by baseline acne severity. Both trials used identical vehicles to maintain blinding, with evaluators masked to treatment assignment. Schleicher et al. [14] used a split-face approach with random allotment of trifarotene to one side of the face and maintained investigator blindness through patient-independent photographic reviews. Blume-Peytavi et al. [12] was unblinded, consistent with the nature of a long-term open-label safety study. Alexis et al. [10] used parallel-group randomization with a matched vehicle cream to preserve blinding of both patients and investigators, with 123 participants randomized across treatment groups (60 trifarotene, 63 vehicle). Randomization and blinding quality for each trial is appraised in Table 1.

Statistical Analyses in Articles 

Tan et al. [5] analyzed categorical endpoints with the Cochran-Mantel-Haenszel test and continuous lesion count changes with analysis of covariance (ANCOVA), adjusting for baseline severity. Missing data were handled using the last-observation-carried-forward (LOCF) imputation method. Del Rosso et al. [13] used mixed-effects models to account for repeated measures and missing data. Schleicher et al. [14] employed within-patient statistical comparisons using paired tests, with evaluator blinding to minimize bias. The long-term safety trial by Blume-Peytavi et al. [12] primarily reported descriptive statistics without formal comparators. Alexis et al. [10] used prespecified between-group comparisons of ODS and PAHPI scores (defined above) at Weeks 12 and 24, with statistical significance defined a priori for each timepoint.

Safety and Tolerability

Across all five trials, trifarotene was generally well tolerated. Adverse events mainly consisted of mild to moderate localized skin reactions typical of topical retinoid therapy, most commonly erythema, dryness, scaling, and transient stinging or burning. These consistently peaked during the first two to four weeks of treatment and then diminished over time.

In the Phase III trials by Tan et al. [5], discontinuations due to local reactions occurred in less than 2% of patients, and tolerability was slightly better on the trunk than on the face.

In the Del Rosso et al. [13] combination trial, rates of dryness and erythema were modestly higher in the trifarotene plus doxycycline arm but remained manageable. Overall, treatment-emergent adverse events occurred in approximately 13% of patients, comparable to the control group rate.

The Schleicher et al. [14] scar-focused study reported similar early-onset tolerability issues, including tightness, erythema, burning, and pruritus, again peaking around Week 2 and improving with emollient or moisturizer use. Discontinuations were rare.

Long-term safety was assessed in Blume-Peytavi et al. [12], in which treatment-related adverse events occurred in about 12%-13% of participants. Almost all events were considered mild to moderate, and the discontinuation rate stayed at 2.9%.

Alexis et al. [10] similarly reported a favourable safety profile across the full range of Fitzpatrick skin types studied (a standard six-category classification of skin tone and sun sensitivity), consistent with the tolerability pattern seen in the other included trials, with no new or unexpected safety signals found in this population.

No systemic adverse events attributable to trifarotene were identified in any of the studies. Taken together, these findings suggest a consistent safety profile characterized by transient, predictable local skin reactions, low discontinuation rates, and sustained tolerability with long-term use; a side-by-side comparison of adverse event rates across all five trials is provided in Table 4.

Short Term Efficacy (12-24 Weeks)

In the Tan et al. [5] Phase III RCTs, trifarotene demonstrated statistically significant improvements over vehicle in IGA and PGA success rates at Week 12, the trial’s primary efficacy endpoints (p < 0.001). The mean percentage reduction in inflammatory lesions exceeded 50% in the treatment arms, with significant reductions also seen in noninflammatory lesions. The trials included over 2,400 patients combined, providing strong statistical power. Trifarotene outperformed vehicle on all primary facial endpoints and secondary truncal endpoints. Facial IGA success rates were 29.4%-42.3% compared to 19.5%-25.7% with vehicle. Truncal PGA success rates were 35.7%-42.6% compared to 25.0%-29.9%. Inflammatory and non-inflammatory lesion counts decreased more with trifarotene (absolute reductions roughly -19 to -30 versus vehicle, all p<0.001).

Del Rosso et al. [13] found that at Week 12, the trifarotene-plus-doxycycline group achieved greater absolute lesion reductions compared to the vehicle-plus-placebo group: total lesions -69.1 vs -48.1 (p < 0.0001), inflammatory lesions -29.4 vs -19.5 (p < 0.0001), and noninflammatory lesions -39.5 vs -28.2 (p < 0.0001). IGA success was the trial’s primary endpoint and was achieved in 31.7% of the trifarotene-plus-doxycycline group compared to 15.8% of the vehicle-plus-placebo group (p=0.0107). Because this trial tested trifarotene combined with doxycycline against vehicle combined with placebo, these results reflect the efficacy of the combination regimen; they should not be read as isolating trifarotene’s independent effect (Table 4).

In Schleicher et al. [14], the split-face design enabled comparisons within each patient, controlling for variance among individuals. Beyond scar outcomes, trifarotene improved active acne measures at Week 24, with IGA success increasing to 63.6% compared to 31.3%, and larger percentage reductions in total (70% vs 45%) and inflammatory (76% vs 48%) lesion counts (all p < 0.0001).

In the Blume-Peytavi et al. [12] open-label long-term study, early efficacy was observed by Week 12, with IGA success of 26.6% (face) and PGA success of 38.6% (trunk).

Alexis et al. [10] evaluated trifarotene specifically for acne-induced hyperpigmentation and produced a more mixed result than the other included trials. At Week 12, ODS scores improved significantly more with trifarotene than vehicle (−1.6 vs −1.1, p=0.03); however, at the trial’s prespecified primary endpoint of Week 24, ODS scores were statistically comparable between groups (−2.1 vs −2.1, p=0.8879), meaning the study did not meet its primary endpoint at that timepoint. Despite this, PAHPI, the secondary hyperpigmentation-specific measure, showed a significantly greater reduction with trifarotene by Week 24 (−18.9% vs −11.3%). This pattern of an early significant effect that did not persist on the primary endpoint, alongside a significant effect on a related secondary measure, is a genuinely mixed finding and is treated as such throughout this review rather than characterized as a straightforward positive result. Full figures are reported in Table 4, with the corresponding bias appraisal in Table 1.

Long Term Efficacy (52 Weeks)

Blume-Peytavi et al. [12] reported that success by Week 52 improved to 65.1% (IGA) and 66.9% (PGA), with combined IGA and PGA success in the same patient of 57.9%. This longer-term data supports sustained benefit and tolerability. No formal statistical comparisons versus control were reported, as this was an open-label, non-comparative study; the corresponding ROBINS-I appraisal, reflecting this lack of a comparator, is presented in Table 2.

Patient-Reported Outcomes 

Patient-reported outcomes in the included trials complemented clinician assessments and demonstrated that clinical improvements were associated with measurable benefits in quality of life.

In Del Rosso et al. [13], patients receiving trifarotene plus doxycycline reported significantly greater improvement on the Acne-QoL scale (mean reduction -30.8 vs -22.6, p<0.05) and higher treatment satisfaction (86% vs 54%) compared to vehicle plus placebo.

Schleicher et al. [14] assessed scar-specific outcomes and found higher rates of patient-reported scar improvement with trifarotene (49% vs 37% reporting “very few scars”), supporting both aesthetic and psychosocial benefit.

In Blume-Peytavi et al. [12], DLQI/CDLQI scores improved steadily, with the proportion of patients reporting “no effect of acne on life” rising from 22.6%-30.9% at baseline to over 53% at Week 52.

The Tan et al. [5] trials did not include patient-reported outcomes, limiting their assessment to clinician-reported efficacy.

Alexis et al. [10] reported high patient satisfaction and adherence to the combined trifarotene and skincare regimen, with patients across the studied Fitzpatrick skin types reporting the treatment approach as acceptable and sustainable over the 24-week course.

Collectively, the trials demonstrated consistent improvements in quality of life and patient satisfaction, reinforcing the clinical relevance of trifarotene beyond lesion reduction. A full summary of patient-reported outcome types and definitions is provided in Table 3.

Integrated Findings 

Taken together, the five included studies (Table 4) suggest that topical trifarotene is generally well tolerated and effective for moderate to severe acne vulgaris. Across randomized trials and one long-term prospective study, trifarotene was associated with improvement in investigator-assessed outcomes, lesion counts, and selected patient-reported measures. The available evidence is especially notable for including dedicated truncal acne data and, more recently, dedicated data on acne-induced hyperpigmentation in a population enriched for darker skin types, although direct comparisons across studies remain limited by differences in design and outcome reporting, and the hyperpigmentation-focused trial’s mixed primary endpoint result tempers an otherwise consistently favourable evidence base.

Discussion 

The available clinical trial evidence supports trifarotene as a useful topical option for the treatment of acne vulgaris. Differences in study design, comparator choice, and measured outcomes make direct comparison between trials more difficult, but they also provide a broader picture of how trifarotene has been studied across facial, truncal, combination-therapy, scar-focused, and long-term settings.

Explanation of Findings

Throughout the review, trifarotene demonstrated clinically meaningful improvements in acne lesions, as measured by IGA, SGA, and PGA success rates and lesion counts (Table 4), alongside more mixed but still clinically informative findings for acne-induced hyperpigmentation. Variability in study design should be considered when interpreting these results. The largest study providing the strongest evidence of efficacy was conducted by Tan et al. [5], which enrolled over 2,400 patients in a large Phase III randomized controlled trial. This study demonstrated strong short-term efficacy, using rigorous randomization and blinding procedures to minimize bias, though its shorter duration means it cannot speak to long-term efficacy on its own.

LOCF and Mixed-Effects Models for Repeated Measures (MMRM)

Tan et al. [5] used LOCF methods to address missing data. This approach assumes that if a patient drops out before the end of Week 12, their status at the time of dropout is carried forward as their final result. This is a conventional and conservative approach, but it can, in principle, overestimate treatment effectiveness if dropouts occur during a period of worsening disease; discontinuation rates in this trial were low (under 2%, Table 4), which limits the practical impact of this assumption. Sensitivity analyses, such as multiple imputation or worst-case scenario analyses, could further strengthen confidence in these estimates in future work.

In contrast, Del Rosso et al. [13] used MMRMs, which use all available data points at each time point and estimate missing values by modeling within-patient trajectories and between-patient variability. This approach makes fewer assumptions about post-dropout outcomes and captures trends over time while adjusting for baseline severity, offering a complementary methodological perspective alongside the Tan et al. [5] findings.

Mixed Regimen Treatments 

Del Rosso et al. [13] evaluated combined trifarotene and doxycycline therapy compared with vehicle and placebo and reported substantial reductions in lesion counts (Table 4). Because the study did not include a trifarotene-only arm, the independent contribution of trifarotene cannot be isolated with certainty from that of doxycycline. This is an important interpretive limitation: the reported effect sizes describe the combination regimen as a whole, not trifarotene monotherapy. Even so, the trial remains clinically relevant because combination therapy is common in moderate to severe acne, and the findings suggest that trifarotene can function effectively within that treatment context.

Measuring Scars Instead of Acne 

Schleicher et al. [14] focused on treating acne scars rather than active acne lesions, providing new evidence regarding the downstream outcomes of trifarotene therapy. This distinction is clinically significant, as scars are a consequence of inadequately controlled acne and are associated with long-term psychosocial burden. The study demonstrated that trifarotene led to measurable improvements in scar counts and patient-reported scar severity (Table 4), while maintaining favourable IGA responses. These findings suggest that trifarotene’s therapeutic utility may extend beyond lesion reduction to managing acne sequelae. Scar-focused studies are inherently more challenging to interpret, however, as the magnitude of improvement is typically smaller than in active acne trials due to the gradual nature of scar remodelling, and scales such as the SGA have limited sensitivity to detect subtle changes over short treatment periods.

Scar outcomes also rely heavily on patient perception, which can introduce reporting bias, particularly when patients know they are receiving active treatment. Combining scar outcomes with IGA findings nonetheless strengthens the case for trifarotene’s broader therapeutic relevance, highlighting its potential to both reduce active disease and mitigate longer-term physical and psychosocial consequences.

*Addressing Hyperpigmentation as an Acne* *Sequela*

Alexis et al. [10] extended the evidence base for trifarotene beyond active lesions and scarring to acne-induced hyperpigmentation, a sequela with particular relevance for patients with darker skin types. The trial’s result is genuinely mixed and is worth interpreting carefully rather than folding into a uniformly positive narrative. The pre-specified primary endpoint, change in ODS at Week 24, was not met, with trifarotene and vehicle producing statistically indistinguishable results at that timepoint (Table 4). At the same time, trifarotene showed a significant early advantage at Week 12 and a significant benefit on PAHPI, the hyperpigmentation-specific secondary measure, by Week 24. One plausible explanation, consistent with the study’s own discussion, is that the structured skincare and photoprotection regimen given to both arms may have narrowed the gap between groups over the full 24 weeks, since sun protection alone can meaningfully improve post-inflammatory hyperpigmentation. This does not overturn the null primary result, but it offers a reasonable interpretive frame for why an early, significant effect did not persist as a primary endpoint difference by Week 24.

This trial’s inclusion strengthens the review in a specific way, as it directly addresses acne-induced hyperpigmentation, a sequela that is common, psychosocially burdensome, and disproportionately affects patients with skin of colour, a population otherwise underrepresented across the other included trials.

Variation in Split-Face Design 

The Schleicher et al. [14] trial employed a split-face design, treating one half of the face with trifarotene and the other with vehicle. This method controls for inter-patient variability in acne severity, skin type, and adherence, enhancing internal validity, and allows for direct assessment of treatment effects even with a modest sample size. This design does introduce some limitations, including potential performance bias, since patients are aware of the treatment allocation, and the possibility of crossover effects if topical agents diffuse to the contralateral side; these considerations are reflected in the “some concerns” rating this trial received in Table 1. Split-face studies also may not fully represent real-world clinical practice, where retinoids are generally used across the entire affected area. Overall, the Schleicher et al. [14] trial offers valuable, well-controlled data on trifarotene’s efficacy and tolerability for scars, complementing the larger parallel-group trials in this review.

Unique Clinical Outcomes in the Long Term

The Blume-Peytavi et al. [12] study provides the only available long-term data on trifarotene, extending treatment to 52 weeks and showing sustained improvements in IGA and PGA success rates, lesion reduction, and patient-reported quality of life. These findings are clinically meaningful, as durable efficacy and tolerability are rarely documented in acne trials, which typically conclude at 12 or 24 weeks. The absence of a comparator arm is a genuine limitation of the design and is reflected in the “serious” overall bias rating in Table 2; without a control, it is difficult to fully separate the drug’s therapeutic effect from the natural course of acne or placebo-type responses. This trial nonetheless provides valuable real-world safety data over a clinically meaningful duration that shorter controlled trials cannot offer.

Differences Between IGA and PGA

Understanding differences in treating facial versus truncal acne is important for clinical practice and recommendations. In the Tan et al. [5] trial, PGA (truncal) success rates (35.7%-42.6%) were numerically similar to, and in this range slightly higher than, IGA (face) success rates (29.4%-42.3%), with both showing highly significant improvement over vehicle (Table 4). The inclusion of truncal endpoints in this study was a unique contribution to its FDA approval.

Del Rosso et al. [13] assessed facial acne only and did not report PGA or truncal outcomes. Schleicher et al. [14] similarly did not report PGA, studying only SGA and facial IGA. Blume-Peytavi et al. [12], notably, found truncal outcomes to be numerically stronger than facial outcomes, suggesting trifarotene may offer a particularly durable long-term benefit for the trunk. Together, these studies help fill a meaningful gap in the understanding of truncal acne treatment.

Patient-Reported Outcomes

A key difference across the trials was the inclusion of patient-reported outcomes. The trials that incorporated patient-reported outcomes consistently showed that clinical improvements translated into measurable gains in quality of life and patient satisfaction, reinforcing that lesion reduction alone understates the clinical value of treatment. The absence of patient-reported outcomes data in the Tan et al. [5] trials is a notable gap given their otherwise central role in the evidence base, and future trials of this kind would benefit from incorporating patient-reported measures from the outset.

Limitations of the Trials

In relation to the broader acne literature, these findings suggest that trifarotene performs comparably to other topical retinoids while offering distinct strengths in truncal disease and long-term tolerability. The absence of direct head-to-head trials means its relative position among retinoids is still an open question rather than an established fact. This review did not identify trials directly comparing trifarotene to other topical retinoids (e.g., adapalene, tretinoin, tazarotene) for acne treatment. As a result, this review focuses on trifarotene’s performance against vehicle rather than ranking it against other retinoids. Establishing its comparative effectiveness and tolerability will require future head-to-head randomized controlled trials or datasets suitable for meta-analysis.

A further consideration is the population enrolled across these trials (summarized in Table 4), which was predominantly adolescents and young adults, consistent with the typical age of onset for acne. This leaves an opportunity for future research to better characterize adult-onset or persistent acne. Similarly, most of the included trials were conducted in academic or urban research centres in North America and Europe with limited reported racial and ethnic diversity, which leaves open questions about generalizability to rural or resource-limited settings. Alexis et al. [10] is a partial exception: approximately 70% of its participants had Fitzpatrick skin types IV-VI, thus providing more direct evidence on trifarotene’s use in patients with darker skin tones than the other included trials offer. This remains an isolated data point rather than a systematic strength of the evidence base, however, and subgroup analyses by skin phenotype in facial and truncal acne trials specifically would still be a valuable addition to future research.

A related limitation is the concentration of sponsorship within this evidence base: all five included trials were funded by Galderma, the manufacturer of trifarotene, and most author teams reported consulting, speaking, or investigator relationships with the company. Industry funding does not by itself indicate biased results, and the risk-of-bias assessments in this review (Tables 1, 2) evaluate methodological rigor independent of funding source. However, because every included trial originates from the same sponsor, this review cannot rule out the possibility of selective publication or a systematically favourable framing of results specific to trifarotene, and independent, non-industry-funded replication would meaningfully strengthen confidence in these findings.

Finally, the structured conditions of clinical trials, including the example of applying treatment to only half the face in the split-face design, may not fully mirror routine clinical practice, which is a reasonable consideration when translating these findings to real-world use.

Limitations of This Review

This review was based on a focused PubMed search. PubMed captures a substantial portion of the dermatology literature, and given the small, well-defined body of trifarotene-specific trial data, this single-database approach was judged sufficient for a narrative synthesis. A systematic review with multiple databases and dual independent screening would be a reasonable next step to further confirm completeness of the evidence base.

Study screening and data extraction were performed by a single reviewer, which is an accepted approach for a narrative review of this scope but differs from the dual-review standard used in formal systematic reviews. Risk-of-bias tools were used to provide a more structured appraisal of study quality within this framework (Tables 1, 2).

Variation in study design, comparators, treatment regimens, follow-up duration, and reported outcomes precluded quantitative pooling of results. For that reason, findings were synthesized narratively rather than through meta-analysis, consistent with the review’s stated scope.

Future Directions

Ongoing research on trifarotene is needed to better understand its clinical utility. Future studies should include additional long-term, blinded, controlled randomized trials to further validate its efficacy and safety, as the current evidence base includes only one long-term study.

Trials that directly compare trifarotene with other retinoids in head-to-head studies are needed to establish a comparative scale of effectiveness. Within the scope of this review, currently published studies compare only the safety and tolerability of different retinoids, not their comparative efficacy in treating acne.

Future studies, even those focused solely on acne, would benefit from more diverse populations and ethnicities. Investigating rural patients and older adults would also improve external validity. Additionally, replication of the hyperpigmentation findings from Alexis et al. [10] in larger, longer-duration trials would help clarify whether the null Week 24 primary endpoint reflects a true plateau in effect or an artifact of the shared skincare regimen used in both study arms, since post-inflammatory hyperpigmentation often persists long after active lesions have healed and can meaningfully affect self-image and quality of life.

Future studies should also integrate clinical effectiveness with real-world effectiveness by addressing adherence, cost, and access to treatment, helping to fill remaining gaps in safety, tolerability, and effectiveness data.

## Conclusions

This review supports trifarotene as a generally well-tolerated and effective topical retinoid for the treatment of moderate to severe acne vulgaris, with a particularly useful evidence base for truncal disease and emerging evidence in acne-induced hyperpigmentation among patients with darker skin types. The available studies suggest meaningful benefit in lesion reduction, investigator-assessed success rates, and selected patient-reported outcomes, alongside an expected and manageable tolerability profile, although the hyperpigmentation-focused trial’s mixed primary endpoint result is a reminder that this newer evidence should be interpreted with appropriate caution rather than folded uncritically into the broader positive narrative. The current literature can be further strengthened by additional trials, broader population diversity, and direct head-to-head comparisons with other topical retinoids. Overall, trifarotene appears to be a valuable addition to acne treatment, especially when truncal involvement, hyperpigmentation risk, or longer-term management is an important consideration.

## References

[1] Tan JK, Bhate K (2015). A global perspective on the epidemiology of acne. Br J Dermatol.

[2] O'Neill AM, Gallo RL (2018). Host-microbiome interactions and recent progress into understanding the biology of acne vulgaris. Microbiome.

[3] Kotekoglu D, Parlakdag A, Koramaz FS (2020). Internalized stigma in acne vulgaris and its relationship with quality of life, general health, body perception, and depression. Niger J Clin Pract.

[4] Zaenglein AL, Pathy AL, Schlosser BJ (2016). Guidelines of care for the management of acne vulgaris. J Am Acad Dermatol.

[5] Tan J, Thiboutot D, Popp G (2019). Randomized phase 3 evaluation of trifarotene 50 μg/g cream treatment of moderate facial and truncal acne. J Am Acad Dermatol.

[6] Thoreau E, Arlabosse JM, Bouix-Peter C (2018). Structure-based design of Trifarotene (CD5789), a potent and selective RARγ agonist for the treatment of acne. Bioorg Med Chem Lett.

[7] Aubert J, Piwnica D, Bertino B (2018). Nonclinical and human pharmacology of the potent and selective topical retinoic acid receptor-γ agonist trifarotene. Br J Dermatol.

[8] Galderma Laboratories. (2019, October 7 (2026). Galderma receives FDA approval for AKLIEF® (trifarotene) cream, 0.005%, the first new retinoid molecule for the treatment of acne in over 20 years. https://www.galderma.com/news/galderma-receives-fda-approval-akliefr-trifarotene-cream-0005-first-new-retinoid-molecule.

[9] Naik PP (2022). Trifarotene: a novel therapeutic option for acne. Dermatol Res Pract.

[10] Alexis A, Del Rosso JQ, Forman S (2024). Importance of treating acne sequelae in skin of color: 6-month phase IV study of trifarotene with an appropriate skincare routine including UV protection in acne-induced post-inflammatory hyperpigmentation. Int J Dermatol.

[11] Haddaway NR, Page MJ, Pritchard CC, McGuinness LA (2022). PRISMA2020: an R package and Shiny app for producing PRISMA 2020-compliant flow diagrams, with interactivity for optimised digital transparency and open synthesis. Campbell Syst Rev.

[12] Blume-Peytavi U, Fowler J, Kemény L (2020). Long-term safety and efficacy of trifarotene 50 μg/g cream, a first-in-class RAR-γ selective topical retinoid, in patients with moderate facial and truncal acne. J Eur Acad Dermatol Venereol.

[13] Del Rosso JQ, Johnson SM, Schlesinger T (2022). A randomized, controlled trial of trifarotene plus doxycycline for severe acne vulgaris. J Clin Aesthet Dermatol.

[14] Schleicher S, Moore A, Rafal E (2023). Trifarotene reduces risk for atrophic acne scars: results from a phase 4 controlled study. Dermatol Ther (Heidelb).

[15] Sterne JA, Savović J, Page MJ (2019). RoB 2: a revised tool for assessing risk of bias in randomised trials. BMJ.

[16] Sterne JA, Hernán MA, Reeves BC (2016). ROBINS-I: a tool for assessing risk of bias in non-randomised studies of interventions. BMJ.

[17] Rao DB, Georgiev EL, Paul PD, Guzman AB (2026). Cyclandelate in the treatment of senile mental changes: a double-blind evaluation. J Am Geriatr Soc.

[18] Layton A, Dréno B, Finlay AY (2016). New patient-oriented tools for assessing atrophic acne scarring. Dermatol Ther (Heidelb).

[19] Savory Savory, S. A., Agim Agim, N. G., Mao Mao, R. R. (2014). Reliability assessment and validation of the postacne hyperpigmentation index (PAHPI), a new instrument to measure postinflammatory hyperpigmentation from acne vulgaris. Journal of the American Academy of Dermatology.

[20] Finlay Finlay, A. Y., & Khan, G. K. (1994). Dermatology Life Quality Index (DLQI)—a simple practical measure for routine clinical use. Clinical and Experimental Dermatology.

[21] Lewis-Jones Lewis-Jones, M. S., & Finlay, A. Y. (1995). The Children’s Dermatology Life Quality Index (CDLQI): initial validation and practical use. British Journal of Dermatology.

[22] Girman Girman, C. J., Hartmaier Hartmaier, S. S., Thiboutot Thiboutot, D. D. (1996). Evaluating health-related quality of life in patients with facial acne: development of a self-administered questionnaire for clinical trials. Quality of Life Research, an international journal of quality of life aspects of treatment, care and rehabilitation.

